# Quality of effort in college activities and learning gains: A case study in China

**DOI:** 10.3389/fpsyg.2022.971639

**Published:** 2022-11-11

**Authors:** Chengmengxue Sun, Jingjing Liang, Ruijun Du, Jiani Ma

**Affiliations:** ^1^Shenzhen Bao’an Education Science Research Institute, Guangdong, China; ^2^Faculty of Education and Social Work, University of Auckland, Auckland, New Zealand; ^3^Faculty of Education, Beijing Normal University, Beijing, China

**Keywords:** learning experiences, learning gains, Chinese undergraduates, College Student Experiences Questionnaire, quality of effort in college activities

## Abstract

This study investigated the mechanism between Chinese undergraduates’ quality of effort in college activities and learning gains, using the Chinese College Student Experiences Questionnaire (CCSEQ). 2,990 undergraduates were recruited at a case Chinese university. Gender, grade, and type of high school differences were found in Chinese undergraduates’ quality of effort in college activities and learning gains. Compared with data from American Norms, Chinese undergraduates have less interaction with teachers, lower classroom participation, and lower intellectual skills gains. Quality of effort in college activities contributed more to learning gains than demographics, with different influences based on the types of college activities. This research verifies the applicability of the CSEQ in the Chinese context and reveals the characteristics of the learning experiences of Chinese undergraduates and the underlying reasons. Recommendations for programme designers, university faculty, and undergraduates themselves were suggested.

## Introduction

In 2021, the gross enrollment rate of higher education in China reached 57.8% (Ministry of Education of the People’s Republic of China, 2022). China has entered the stage of popularization of higher education, and educational demands have gradually shifted from the pursuit of quantity to quality. In this trend, undergraduates expect to reap the benefits of high-quality higher education, with increasing attention has been paid to their learning experiences and what they can gain from universities. These desires have become important indicators of deciding undergraduates’ learning satisfaction and university enrolment. With this in mind, many universities emphasize improving undergraduates’ learning experiences and gains.

Focusing on undergraduates’ learning experiences and intensive development is also an urgent requirement for the current educational reform in China. Traditionally, Chinese universities have emphasized teaching over learning and outcomes over processes. As an important indicator of the quality of higher education, the learning experiences of undergraduates are often forgotten or neglected. It is either obscured by figures such as the number of students and the teacher-student ratio in universities, or simplified to academic performance (Zhou and Zhou, 2007). In this light, Chinese scholars and universities have increasingly introduced new concepts or instruments, such as the College Student Experiences Questionnaire (CSEQ), to respond to this tendency that respects students’ dominant position and discourse rights, and advocates that the evaluation subject shift from external stakeholders to students, and evaluation criteria should shift from outcomes-oriented to process-oriented (Chen, 2009).

To date, many studies have conducted empirical studies using CSEQ in the Chinese context (Bai and Zhou, 2018) and mainly in the United States (Gonyea et al., 2003; Kuh, 2009). Research has found that demographics, quality of effort in college activities, and institutional environment had a significant impact on undergraduates’ learning gains. However, few studies focus on their learning experiences from a comparative perspective. For example, what are the potential differences and similarities between Chinese and American undergraduates’ learning experiences? and what practical implications can we propose based on the gap in their learning experiences? Answering these questions could benefit undergraduates and programme designers in higher education institutions (HEIs) at home and abroad.

It is undeniable that there are some differences in learning experiences between China and the United States. For example, most Chinese undergraduates spend much time in Party and League activities compared to their American counterparts, which may affect their learning gains. In addition, the teaching mode tends to be teacher-centered in Chinese universities, which may lead to differences in teacher-student interaction, learning initiative, curriculum design, and teaching management compared to the American teaching mode. Nevertheless, most learning experiences of Chinese and American students in college are similar, as verified by previous studies conducted in the Chinese context (Chi et al., 2017) that undergraduates in both countries use various campus facilities, devote themselves to course learning, and interact with classmates and teachers, which are the variables that are commonly measured by the CSEQ questionnaire. Therefore, a comparative study of undergraduates’ experiences between China and the United States is applicable.

To fill the above gaps, this study uses a representative case of Chinese undergraduate students at university B (mainland China). University B was selected because of its long-standing commitment to education and teaching reform with a focus on undergraduates’ learning experiences and gains. Therefore, taking university B as an example, we conducted this research to understand undergraduates’ learning experiences and learning gains. Based on the potential findings, university B will apply them to improve practices and services to enhance the quality of education and governance further, so as to improve students’ learning experiences, enhance the institutional reputation, and generalize successful experiences to similar institutions.

In summary, this study aims to reveal the characteristics of Chinese undergraduates’ learning experiences and gains, explore the associated influential mechanisms, and identify the similarities and differences compared to American undergraduates, with three overarching questions:

1. What are the characteristics of the quality of effort in college activities and learning gains of Chinese undergraduates?
2. Is there any difference between the quality of effort in college activities and learning gains of Chinese undergraduates and American undergraduates?
3. What is the strongest predictor of Chinese undergraduates’ learning gains?

## Literature review

### The college student experiences questionnaire

The CSEQ includes three aspects: (a) quality of effort in college activities, (b) college environment, and (c) learning gains. The scale of quality of effort in college activities covers the students’ course learning, student acquaintance, library experiences, science and quantitative experiences, writing experiences, personal experiences, experiences with faculty, activities using campus facilities, computer and information technology, and experiences with art, music and theater, clubs, and organizations. The scale of the college environment is used to reflect students’ perceptions of the college environment and interpersonal relationships related to personal development. The scale of learning gains covers five areas: vocational preparation, general education, intellectual skills, personal development, and science and technology. Personal development means that students form their values and moral judgment standards, learn to get along with others, and have strong adaptability. Science and technology mean that students know the nature and development of science and technology. General education refers to general knowledge and aesthetic ability. Vocational preparation refers to having the knowledge and ability to engage in a profession. Intellectual skills mean that students have good language expression, writing skills, systematic thinking, and logical analysis. The theoretical basis of CSEQ is Pace’s quality of effort model, assuming that the quality of effort in the different types of college activities is highly correlated with relevant learning gains (Pace, 1984), satisfaction, and persistence. Students will benefit from the time and energy engaged in meaningful educational activities (Hakes, 2010; Utami et al., 2015).

Research on subgroups’ learning experiences is one of the hotspots. Although the impact of demographics was lower than the quality of effort in college activities and the college environment (Watson and Kuh, 1996), researchers have found that background factors cannot be ignored. Grade and other factors have a significant impact on students’ learning gains (Pascarella and Terenzini, 1991; Cvencek et al., 2014; Hu, 2018). Sümer et al. (2012) found that Turkish doctoral students had a low quality of effort in course learning and personal development. Based on black and white students’ learning experiences, Bista (2013) found that academic level, length of stay, and original country significantly predicted learning gains: graduate students, students from South-Central Asia to Southeast Asia, and students staying more than 2 years had higher learning gains than undergraduate students, East Asian students, and students with a stay of 1 year or less. Yeh (2004) also surveyed Asian international graduate students and found that time spent in the United States, relationships with faculty, and awards (scholarships and bursaries) were highly related to what they gained. Given those different conclusions from participants with diverse demographics, Chinese undergraduates’ learning experiences, who have different demographics, may differ from their counterparts in other jurisdictions, which is quite meaningful to explore.

Concerning Chinese undergraduates’ learning experiences, Chi et al. (2017) found that student engagement in college activities played a mediating role in the college environment and intellectual development. Their interactions with faculty could effectively predict their intellectual development. In Hong Kong, Tam (2004) found that students achieved significant gains in intellectual and personal development, but their gains in vocational preparation were limited. The quality of effort in college activities and interaction with the environment affected students’ cognitive and emotional gains, but demographics had limited influence on the undergraduates’ learning experiences. Chen (2009) compared the experiences of Chinese students with Japanese students and noted that Chinese students’ quality of effort in course learning was the highest, but their interactions with the faculty were the lowest. Chinese students had higher gains in science and technology, intellectual skills, and personal development, and fewer gains in the other aspects. However, the number of empirical studies in the Chinese context is relatively limited (Chi et al., 2017), especially from a comparative perspective. Therefore, this study aims to explore the effect of Chinese undergraduates’ quality of effort in college activities on learning gains, and what are the gaps between Chinese and American undergraduates’ learning experiences.

### Quality of effort in college activities and learning gains

Quality of effort in college activities is widely regarded as a critical component of student learning and development. It is defined as “how often, during the current school year, students engage in various activities related to the use of campus facilities and opportunities intended for their learning and development” (Trowler, 2010), namely, the frequency students engage in college activities (Graham et al., 2007). Different types of structures were proposed. Fredricks et al. (2004) proposed a three-component model of student engagement from cognitional, emotional and behavioral perspectives. The National Survey of Student Engagement (NSSE) (2010) summarized student engagement into academic challenges, active learning, student-faculty interactions, educational experiences, and learning environment. Student Experience in the Research University (SERU) focused on research universities, and emphasized students’ scientific research ability, and academic participation. The CSEQ defines students’ quality of effort in college activities as students’ engagement in multiple activities: library experiences, computer and IT experiences, course learning experiences, writing experiences, art, music and theater experiences, campus facility experiences, personal experiences, science and quantitative experiences, clubs and organizations experiences, experiences with faculty, experiences with student acquaintances, and so on. This definition is more detailed than others, and is close to college students’ real life and experiences.

Learning gains refer to the progress that students make in areas that are important to their growth and education (Hayek, 2001), based on students’ reflections on their college experiences and personal growth. Fulks believes that learning gains are specific and measurable goals and results that students are expected to achieve in learning (Fulks, 2009). It is the ability of students to demonstrate their knowledge, skills and values after completing a series of courses or development projects (Kuh and Hu, 2001). Astin A. (1993) proposed an Input-Environment-Output (I-E-O) model and explained that student demographics, learning engagement, and school environment together influence learning gains. Compared to the Australasian Survey of Student Engagement (AUUSE), which measures learning gains by higher-order thinking, general learning outcomes, career readiness, grades, departure intentions, and satisfaction, CSEQ measures it by personal development, science and technology, general education, vocational preparation, and intellectual skills. This type of measurement is comprehensive and can reflect the holistic development of students as a whole person.

Exploring the relationship between the quality of effort in college activities and learning gains could provide empirical feedback on the quality of higher education. Research has shown that undergraduates’ involvement in college activities greatly affects their academic achievements (Zilvinskis et al., 2015), the rate of dropouts (Astin, 1975), and educational gains (Alnusair, 2000; Miller, 2012). Additionally, the type of college activities that undergraduates participate in play different roles in learning gains (Weber et al., 2013). However, no significant relationship between college activities and learning gains is found by others (Hagel et al., 2011). McCormick (2009) and Kuh (2009) hardly indicated that the quality of effort in college activities contributed to students’ success. The key factor affecting students’ academic achievement was their ability, rather than their college experiences (Grayson, 1999). No consensus has been found yet. Let alone the relationship between quality of effort in college activities and learning gains in the Chinese context.

## Materials and methods

### Participants

This study was conducted at university B, a comprehensive university ranked among the top universities in China and globally. This case university is selected because university B owns a well-developed curriculum, highly qualified faculty, and rich facilities. As mentioned earlier, university B places great importance on understanding the learning experiences from the perspective of students, and could provide students with different college activities, which could cover the typologies of college activities measured in the CSEQ scale. Meanwhile, university B offers programs in different types of disciplines at the undergraduate, master, and doctoral levels. The potential results are able to reflect the learning experiences of students from different disciplinary backgrounds.

In May 2018, a total of 3,500 questionnaires were achieved through delivering questionnaires with the assistance of University B administrators. A total of 2,990 valid questionnaires were reached, with a high response rate (85.4%), after removing invalid responses, such as all items being the same response or having too short a response time (e.g., 2 s for one item). Specifically, there were 669 freshmen (22%), 824 sophomores (28%), 858 juniors (29%), and 638 seniors (21%). There were more female (76%) than male respondents. A total of 1,608 undergraduates graduated from provincial key high schools (i.e., top high school in a specific province, 54%), 970 students from county/district key high schools (i.e., good high school in a region, 32%), 388 students from ordinary high schools (i.e., average-level high school, 13%), and 24 students from other types of high schools (1%).

### Measurement

The CSEQ has been applied in American universities with adequate construct validity and reliability. To evaluate college student experiences, the CSEQ was first developed by Pace at the University of California Los Angeles in the 1970s and introduced as a multi-institutional survey tool in 1979. It has been revised three times since the second edition in 1983, the third edition in 1990, and the fourth edition 1998. Pace and Kuh subsequently co-authored the fourth and current edition of the CSEQ (Gonyea et al., 2003).

The CCSEQ was compiled based on the fourth edition of the CSEQ and authorized by Kuh at Indiana University in the 1990s. The CCSEQ was developed using a translation-back translation process. After translating the questionnaire into Mandarin, experts were asked to conduct a back-translation, then the equivalence between the original and translated versions was compared, and finally, a minor revision was conducted to reach an agreement. The original items of the questionnaire were basically retained. Although some items were added, these additional items considered the characteristics of Chinese undergraduates and did not change the original questionnaire. Based on I-E-O (Input-Environment-Output) theory Astin A.W. (1993), the study took into account the importance of the prior experience of undergraduates, and added the type of high school to reflect different levels of education resource allocation and the level of education quality that Chinese undergraduates could have in high schools.

*Independent variables* were the quality of effort in college activities, which included activities in computer and information technology (QECOMPUT), library experiences (QELIB), art, music and theater (QEAMT), science and quantitative experiences (QESCI), personal experiences (QEPERS), experiences with faculty (QEFAC), writing experiences (QEWRITE), campus facility (QEFACIL), clubs and organizations (QECLUBS), course learning (QECOURSE), and student acquaintance (QESTACQ). Specifically, the CSEQ asks how often the student has done or experienced a particular event during the current school year. Four-point balanced rating scale was used (1 = never, 2 = occasionally, 3 = often, and 4 = very often). The higher the score was, the more undergraduates engaged in the activities. A confirmatory factor analysis (CFA) using Mplus (version 8.4) was used. Results indicated that the scale of quality of effort in college activities had good fit characteristics (CFI = 0.94, TLI = 0.92, RMSEA = 0.08, and SRMR = 0.04) and construct validity.

*Dependent variables* were learning gains, which were summarized into gains in personal development (PERSDEV), science and technology (SCITECH), general education (GENED), vocational preparation (VOCPREP), and intellectual skills (INTELSK). Four-point balanced rating scales were used with two negative response points (1 = few, 2 = some) and two positive response pointes (3 = quite a bit, 4 = very much). Table 1 lists the composite reliability (CR) for each of the scales in our sample. All reliabilities were acceptable and good. The model fitted well (CFI = 0.99, TLI = 0.98, RMSEA = 0.07, and SRMR = 0.01).

**Table 1:** Correlations for scale variables.

Variables	1	2	3	4	5	6	7	8	9	10	11	12	13	14	15	16
1. QELIB	0.60															
2. QECOMPUT	0.46	0.55														
3. QECOURSE	0.50	0.56	0.54													
4. QEFAC	0.41	0.42	0.52	0.64												
5. QEWRITE	0.44	0.48	0.52	0.51	0.63											
6. QEFACIL	0.26	0.30	0.39	0.29	0.36	0.58										
7. QEAMT	0.30	0.30	0.36	0.43	0.32	0.22	0.76									
8. QEPERS	0.32	0.38	0.43	0.41	0.44	0.40	0.41	0.60								
9. QECLUBS	0.23	0.28	0.34	0.42	0.31	0.33	0.38	0.40	0.65							
10. QESCI	0.20	0.37	0.30	0.43	0.35	0.23	0.19	0.27	0.27	0.75						
11. QESTACQ	0.32	0.44	0.52	0.42	0.46	0.47	0.35	0.57	0.45	0.32	0.55					
12. PERSDEV	0.26	0.33	0.43	0.31	0.39	0.43	0.24	0.39	0.30	0.24	0.51	0.71				
13. SCITECH	0.15	0.31	0.25	0.37	0.31	0.28	0.16	0.21	0.20	0.63	0.30	0.45	0.81			
14. GENED	0.35	0.24	0.40	0.37	0.35	0.31	0.43	0.34	0.31	0.14	0.36	0.51	0.31	0.68		
15. VOCPREP	0.26	0.26	0.38	0.31	0.32	0.38	0.19	0.27	0.21	0.28	0.34	0.56	0.40	0.53	0.74	
16. INTELSK	0.31	0.39	0.46	0.39	0.47	0.44	0.26	0.37	0.30	0.40	0.47	0.76	0.63	0.57	0.59	0.63
Mean	2.52	2.77	2.58	1.91	2.56	2.85	2.15	2.62	2.20	2.13	2.80	3.05	2.46	2.53	2.85	2.81
SD	0.57	0.55	0.48	0.56	0.62	0.55	0.72	0.57	0.68	0.72	0.50	0.61	0.79	0.64	0.67	0.57
CR	0.82	0.83	0.82	0.91	0.81	0.82	0.89	0.82	0.80	0.91	0.86	0.84	0.89	0.86	0.78	0.83

SD, standard deviation; CR, composite reliability; QELIB, Library Experiences; QECOMPUT, Computer and Information Technology; QECOURSE, Course Learning; QEWRITE, Writing Experiences; QEFAC, Experiences with Faculty; QEAMT, Art, Music, and Theater; QEFACIL, Campus Facilities; QECLUBS, Clubs and Organizations; QEPERS, Personal Experiences; QESTACQ, Student Acquaintances; QESCI, Scientific and Quantitative Experiences; PERSDEV, Personal Development; SCITECH, Science and Technology; GENED, General Education; VOCPREP, Vocational Preparation; and INTELSK, Intellectual Skills.

The figures included on the diagonal are square roots of the AVE. The figures on the triangle elements are correlations among the variables. All variables were significantly correlated (*p* < 0.001).

*Control variables* included gender, grade, and type of high school. These factors were added because we wish to respond to the controversy in the previously mentioned existing studies about whether demographic variables affect undergraduates’ learning gains. Gender (0 = female, 1 = male) was measured as dichotomous dummy variables. Four-point scales were used to measure grade (1 = freshmen, 2 = sophomore, 3 = junior, and 4 = senior) and the type of high school (1 = provincial key level, 2 = county/district key level, 3 = ordinary, and 4 = others). These variables were measured as categorical variables and converted to dummy variables, respectively. Different types of high schools mean different educational resources, and quality of education in the Chinese context. Specifically, provincial key high schools are under the supervision of provincial education authorities, with rich educational resources, high-quality teachers, and relatively better academic performance and learning atmosphere for students. County/district key high schools are under the supervision of regional education authorities, with fewer resources than provincial key high schools but much more educational support than the rest of the two types of high schools.

### Data analysis

After the CFAs with good construct validity, descriptive statistics for all relevant variables were calculated as an initial analysis. To compare the differences in quality of effort in college activities and learning gains between Chinese and American undergraduates, a one-sample *t*-test was used to compare the results of original sample in this study and the CSEQ Fourth Edition American Norms (Gonyea et al., 2003).

One-way ANOVA was used to analyze the gender, grades, and types of high school differences in the quality of effort in college activities and learning gains. Finally, blocked hierarchical regression analysis examined the relationship between the undergraduates’ quality of effort in college activities and learning gains after controlling for demographics.

## Results

### Characteristics of Chinese undergraduates’ experiences

Table 2 shows the characteristics of Chinese undergraduates’ quality of effort in college activities; Chinese undergraduates had the highest level of involvement in QEFACIL (Mean = 2.85, SD = 0.55), followed by QESTACQ (Mean = 2.80, SD = 0.50). Their involvement in QESCI (Mean = 2.13, SD = 0.72) and QEAMT (Mean = 2.15, SD = 0.72) were relatively low, and QEFAC was the lowest (Mean = 1.91, SD = 0.56). In terms of learning gains, the undergraduates scored highest on PERSDEV (Mean = 3.05, SD = 0.61) and lowest on SCITECH (Mean = 2.46, SD = 0.79). These results show that Chinese undergraduates often use college facilities and frequently communicate with acquaintances. But they were less involved in science and art activities and interacted less with teachers. They gained the most in personal development, but the least in science and technology.

**Table 2:** Differences in quality of effort in college activities and learning gains of Chinese undergraduates.

	**Gender (T)**	**Grade (F)**	**High School (F)**
*Quality of effort in college activities*			
QELIB	−6.68***	24.06***	3.29*
QECOMPUT	−4.69***	46.35***	5.21**
QECOURSE	−6.89***	12.14***	12.75***
QEFAC	4.14***	30.56***	3.24*
QEWRITE	−3.14**	83.27***	4.39**
QEFACIL	−2.31*	7.26***	4.67**
QEAMT	−4.79***	2.34	11.36***
QEPERS	−7.48***	4.16**	3.46*
QECLUBS	−0.74	5.11**	2.98*
QESCI	11.52***	3.21*	4.12**
QESTACQ	−3.85***	1.54	9.99***
*Learning gains*			
PERSDEV	−0.61	2.05	2.87*
SCITECH	9.66***	3.88**	1.77
GENED	−0.22	4.22**	2.42
VOCPREP	2.09*	1.77	1.87
INTELSK	3.04**	7.66***	5.74**

QELIB, Library Experiences; QECOMPUT, Computer and Information Technology; QECOURSE, Course Learning; QEWRITE, Writing Experiences; QEFAC, Experiences with Faculty; QEAMT, Art, Music, and Theater; QEFACIL, Campus Facilities; QECLUBS, Clubs and Organizations; QEPERS, Personal Experiences; QESTACQ, Student Acquaintances; QESCI, Scientific and Quantitative Experiences; PERSDEV, Personal Development; SCITECH, Science and Technology; GENED, General Education; VOCPREP, Vocational Preparation; and INTELSK, Intellectual Skills.

* *p* < 0.05;

** *p* < 0.01;

*** *p* < 0.001.

In terms of gender differences, Table 2 indicates gender differences in the quality of effort in college activities except for QECLUBS (*t* = −0.74, *p* > 0.05). Male undergraduates were engaged more in QEFAC and QESCI than female undergraduates, while female undergraduates engaged more in other activities (e.g., QECOURSE, QELIB). In terms of learning gains, male undergraduates gained more than female undergraduates in SCITECH, VOCPREP and INTELSK. These findings imply that male undergraduates interacted more with teachers and were more involved in science activities, while female undergraduates were more involved in classroom activities, library, etc. Male undergraduates gained more knowledge in science and technology, were better prepared for their vocation, and had higher intellectual skill development.

Regarding grade differences, undergraduates with different grades had significant grade differences in the quality of effort in college activities, except for QESTACQ and QEAMT (see Table 2). Senior students engaged more than others in QELIB, QEFAC, and QEWRITE, while freshman students engaged more in QECLUBS and QEFACIL. Freshmen engaged less than others in QECOURSE, QECOMPUT, and QESCI. This means that seniors interacted more with faculty, were more involved in writing activities, and used the library more frequently. Freshmen, on the other hand, were more involved in club activities and use of campus facilities, they were less engaged in course learning, less involved in computer and information technology, and less involved in scientific activities. In terms of learning gains, undergraduates with different grades had statistically significant differences in SCITECH, GENED, and INTELSK. Senior students gained more than others in SCITECH. Freshmen gained less than others in INTELSK. This indicates that seniors achieved greater gains in science and technology, while freshmen had relatively less development in intellectual skills.

Concerning the type of high school differences, undergraduates from different high schools differed in the quality of effort in college activities. Undergraduates from provincial key high schools engaged more than those from other types of high schools in QECOMPUT, QECOURSE, QEWRITE, QEAMT, QESCI, and QESTACQ. They engaged more than students from ordinary high schools in QECLUBS, QEPERS, and QELIB. This indicates that undergraduates from provincial key high schools are more involved in computer and information technology activities, course learning, writing, science and art activities, and interactions with student acquaintances than undergraduates from other types of high schools, and are more involved in club activities, library activities, and activities related to their personal experiences than undergraduates from ordinary high schools. In terms of learning gains, there were statistically significant differences among undergraduates from different types of high schools in PERSDEV and INTELSK. Undergraduates from provincial key high schools gained more than those from district key high schools and ordinary high schools. This means that undergraduate students from provincial key high schools had higher levels of personal development and intellectual skills.

### Differences in learning experiences between Chinese undergraduates and American undergraduates

Compared with the results of American norms (Gonyea et al., 2003), the Chinese sample reported a higher quality of effort in QELIB, QECOMPUT, QEFACIL, QEAMT, QEPERS, QESTACQ, and QECLUBS, and reported lower quality of effort in QECOURSE, QEWRITE, and QEFAC. Except for QEWRITE, QEAMT, and QEPERS, the differences between the Chinese sample and American norms are medium (*d* > 0.40) and large (*d* > 0.60; Hattie, 2009). The results show that compared with the U.S. norms, Chinese undergraduates were more engaged in using the library, computer information technology, and campus facilities, more involved in club and student acquaintances, but less engaged in course learning and interactions with faculty. Regarding the learning gains scales, the Chinese sample reported higher learning gains in PERSDEV, SCITECH, and GENED but lower gains in INTELSK. This means that compared to the U.S. norms, Chinese undergraduates gained more in personal development, science and technology, and general education, but less in intellectual skill development (Table 3).

**Table 3:** Differences in learning experience between Chinese and American undergraduates.

	**Chinese sample**		**American norms**		**One-sample T-test**	**Effect size**
Scale or Factor	Mean	*SD*	Mean	*SD*	*T*	Cohen’s d
*Quality of effort in college activities*						
QELIB	2.52	0.57	2.05	0.58	45.18***	0.81^a^
QECOMPUT	2.77	0.55	2.47	0.59	29.65***	0.52
QECOURSE	2.58	0.48	2.94	0.52	−41.32***	0.71 ^a^
QEFAC	1.91	0.56	2.26	0.62	−34.62***	0.58
QEWRITE	2.56	0.62	2.68	0.61	−10.72***	0.20
QEFACIL	2.85	0.55	2.17	0.64	67.71***	1.10 ^a^
QEAMT	2.15	0.72	2.05	0.75	7.56***	0.13
QEPERS	2.62	0.57	2.46	0.66	15.15***	0.25
QECLUBS	2.20	0.68	1.81	0.81	31.43***	0.50
QESCI	2.13	0.72	2.13	0.77	−0.34	0
QESTACQ	2.80	0.50	2.53	0.69	29.67***	0.42
*Learning gains*						
PERSDEV	3.05	0.61	2.95	0.65	9.35***	0.16
SCITECH	2.46	0.79	2.37	0.81	5.94***	0.11
GENED	2.53	0.64	2.40	0.67	11.16***	0.17
VOCPREP	2.85	0.67	2.84	0.70	0.90	0.01
INTELSK	2.81	0.57	2.94	0.60	−12.63***	0.20

SD, Standard Deviation; a Values represent large effect sizes; QELIB, Library Experiences; QECOMPUT, Computer and Information Technology; QECOURSE, Course Learning; QEWRITE, Writing Experiences; QEFAC, Experiences with Faculty; QEAMT, Art, Music, and Theater; QEFACIL, Campus Facilities; QECLUBS, Clubs and Organizations; QEPERS, Personal Experiences; QESTACQ, Student Acquaintances; QESCI, Scientific and Quantitative Experiences; PERSDEV, Personal Development; SCITECH, Science and Technology; GENED, General Education; VOCPREP, Vocational Preparation; and INTELSK, Intellectual Skills.

*** *p* < 0.001.

### The impact of demographics, quality of effort in college activities on learning gains

Table 4 indicates the impact of demographics and quality of effort in college activities on learning gains. Regarding demographic variables, male undergraduates gained more than females in all types of learning gains. The following section will discuss the impact of demographics and quality of effort in college activities on each learning gain.

**Table 4:** Hierarchical linear regression of gains factors.

**Independent variables**	**PERSDEV**		**SCITECH**		**GENED**	
	**Model 1**	**Model 2**	**Model 1**	**Model 2**	**Model 1**	**Model 2**
**Control variables**						
Grade 1	−0.04	0.01	−0.08**	−0.01	−0.04	0.02
Grade 2	−0.01	0.02	−0.07**	−0.02	−0.03	0.02
Grade 3	−0.05*	−0.01	−0.03	0.01	−0.08***	−0.01
Gender	−0.01	0.05**	0.18***	0.07***	−0.01	0.07***
High School 1	0.07**	−0.01	0.02	−0.02	0.07**	−0.01
High School 2	0.03	−0.02	−0.01	−0.02	0.05	0.00
High School 3	0.01	0.01	−0.02	−0.01	0.02	−0.00
**Predictors**						
QELIB		−0.01		−0.04*		0.13***
QECOMPUT		0.02		0.06**		−0.08***
QECOURSE		0.15***		−0.03		0.14***
QEFAC		−0.04		0.08***		0.07**
QEWRITE		0.10***		0.04*		0.09***
QEFACIL		0.19***		0.13***		0.09***
QEAMT		0.00		0.01		0.25***
QEPERS		0.06**		−0.05**		0.03
QECLUBS		0.02		−0.05**		0.04*
QESCI		0.01		0.53***		−0.08***
QESTACQ		0.27***		0.07***		0.08***
*F*	2.26*	88.85***	16.37***	127.21	2.91**	75.68***
*R* ^2^	0.01	0.35	0.04	0.44	0.01	0.31
Δ*R*^2^	0.01	0.35	0.04	0.40	0.01	0.31
Δ*F*	2.26*	143.20***	16.37***	190.47***	2.91**	121.16***
Durbin-Watson		1.99		2.05		1.98
**Independent variables**			**VOCPREP**	**INTELSK**	**VOCPREP**	**INTELSK**
			**Model**	**Model 2**	**Model 1**	**Model 2**
**Control variables**						
Grade 1			−0.02	0.03	−0.11***	−0.02
Grade 2			−0.00	0.03	−0.04	0.02
Grade 3			−0.04	−0.00	−0.07**	−0.01
Gender			0.04	0.05**	0.06**	0.08***
High School 1			0.06	−0.00	0.11***	0.02
High School 2			0.05	0.02	0.05	0.01
High School 3			0.03	0.03	0.02	0.02
**Predictors**						
QELIB				0.05**		0.02
QECOMPUT				−0.03		0.04
QECOURSE				0.17***		0.14***
QEFAC				0.06*		0.00
QEWRITE				0.06**		0.16***
QEFACIL				0.22***		0.20***
QEAMT				−0.00		0.02
QEPERS				0.00		0.01
QECLUBS				−0.03		−0.00
QESCI				0.11***		0.17***
QESTACQ				0.08***		0.15***
*F*			2.11*	52.73***	7.33***	108.82***
*R* ^2^			0.01	0.24	0.02	0.40
ΔR^2^			0.01	0.24	0.02	0.38
ΔF			2.11*	84.53***	7.33***	170.49***
Durbin-Watson				1.96		1.94

“Gender” means “0 = female,1 = male”; “Grade 1” means “Freshmen” vs. “Senior,” “Grade 2” means “Sophomore” vs. “Senior,” “Grade 3” means “Junior” vs. “Senior”; “High School 1” means “Provincial key level” vs. “Ordinary,” “High School 2” means “County key level” vs. “Ordinary,” “High School 3” means “Others” vs. “Ordinary”; QELIB, Library Experiences; QECOMPUT, Computer and Information Technology; QECOURSE, Course Learning; QEWRITE, Writing Experiences; QEFAC, Experiences with Faculty; QEAMT, Art, Music, and Theater; QEFACIL, Campus Facilities; QECLUBS, Clubs and Organizations; QEPERS, Personal Experiences; QESTACQ, Student Acquaintances; QESCI, Scientific and Quantitative Experiences; PERSDEV, Personal Development; SCITECH, Science and Technology; GENED, General Education; VOCPREP, Vocational Preparation; and INTELSK, Intellectual Skills.

* *p* < 0.05;

** *p* < 0.01;

*** *p* < 0.000

In terms of personal development, model 2 explained 35% of the variance in personal development, which was much higher than the contribution of model 1. QESTACQ (β = 0.27, *p* < 0.001), QEFACIL (β = 0.19, *p* < 0.001), QECOURSE (β = 0.15, *p* < 0.001), QEWRITE (β = 0.10, *p* < 0.001), and QEPERS (β = 0.06, *p* < 0.01) were significant predictors of personal development. The impact of QESTACQ was the highest (β = 0.27, *p* < 0.001). It indicated that undergraduates devoted more effort to student acquaintances, campus facilities, course learning, writing and personal experiences, and gaining more personal development.

In terms of science and technology, demographics only accounted for 4% of the variance, while model 2, adding quality of effort in college activities, explained 44% of the variance. Nine variables of quality of effort in college activities were statistically significant in predicting gains in science and technology, except for QECOURSE and QEAMT. The best predictor of science and technology gain was QESCI (β = 0.53, *p* < 0.001). Therefore, undergraduates invested more effort in science and technology activities, and they gained more in science and technology.

Regarding general education, ten variables of quality of effort in college activities were statistically significant in predicting general education gain, except for QEPERS. The best predictor of general education was QEAMT (β = 0.25, *p* < 0.001), followed by QELIB (β = 0.13, *p* < 0.001), and QECOURSE (β = 0.14, *p* < 0.001). The more effort undergraduates devoted to QEAMT, QELIB, and QECOURSE, the more gains in general education.

With regard to vocational prepare, seven variables of quality of effort in college activities (QELIB, QECOURSE, QEFAC, QEWRITE, QEFACIL, QESCI, and QESTACQ) significant predicted vocational preparation gain, especially QEFACIL (β = 0.22, *p* < 0.001). Undergraduates invested more effort in campus facilities, and they were more likely to gain more in vocational preparation.

As for intellectual skills, five variables of quality of effort in college activities significant influenced intellectual skills gain, which were QECOURSE (β = 0.14, *p* < 0.001), QESCI (β = 0.15, *p* < 0.001), QESTACQ (β = 0.17, *p* < 0.001), QEWRITE (β = 0.17, *p* < 0.001), and QEFACIL (β = 0.20, *p* < 0.001). QEFACIL was the best predictor of intellectual skills gain. Undergraduates devoted more effort to these activities and gained more intellectual skills.

## Discussion

Through a large-scale survey of Chinese undergraduates’ experiences, this study reveals the characteristics of Chinese undergraduates’ quality of effort in college activities and learning gains, and enriches research on learning experiences with a comparative perspective. This section discusses key findings, implications for theory and practice in undergraduate’s development and limitations.

In terms of the characteristics of Chinese undergraduates’ quality of effort in college activities, they were highly involved in activities related to college facilities and student acquaintances. This finding coincides with other studies in China: undergraduates are most involved in activities related to college facilities (Zhang, 2016). The uniform management of undergraduates led them to engage more in activities with college facilities and student acquaintances. Additionally, this study found that Chinese undergraduates were less likely to engage in activities with faculty. One possible explanation is that students’ motivational, emotional, and behavioural engagement in activities is triggered by their previous experiences. Suppose undergraduates experience a supportive institutional climate (Vieno et al., 2005), characterized by adequate material resources, positive faculty-student relationships, or peer support; in that case, they will gain a sense of belongingness and are more likely to engage in relevant activities and maintain professional relationships with faculty or peers constantly. However, studies have demonstrated that the interactions between teachers and students in Chinese universities are inadequate and unequal. Teachers played the “authority” role, and only a few people in the classroom could benefit from the interaction (Li, 2010), which negatively influenced undergraduates’ relational experience and cognition and further constrained their constant involvement in relationship engagement and maintenance. In order to improve the effectiveness of teachers-students interaction, teachers should promote students’ initiative, and create a democratic and consultative atmosphere. Moreover, Chinese undergraduates were less involved in science and art activities. This may result from exam-oriented education evaluation focusing more on academic performance (Zhou and Zhang, 2019); thus, Chinese undergraduates lack interest in participating in other scientific and cultural activities. Based on this, HEIs should reform educational evaluation to encourage undergraduates to actively participate in diverse university activities, broaden their knowledge, improve their scientific and cultural literacy, and achieve whole-person development.

As for learning gains, we found that Chinese undergraduates gain less in general education and science and technology. This result was supported by Wang and Xie (2015) that general education was insufficiently developed in China, which cannot shake the dominance of professional education in practice (Zhang, 2014). In addition, China’s general education curriculum lacks an overall design, which mainly arranges courses according to the characteristics of university teachers and fails to cover scientific and cultural fields. This may lead to fewer courses in science and technology in some universities (Li et al., 2001), and undergraduates’ learning gains in science and technology are not enough. Thus, we suggest that Chinese scholars and program designers further clarify general education concepts and objectives, improve the curriculum design, and allow students to learn through a personalized instructional environment. Meanwhile, teachers should be qualified with diverse disciplinary knowledge, and the ability to adapt the learning material, track students’ development, and communicate and collaborate with students (Troussas et al., 2018).

The study found significant gender, grade, and high school type differences in the quality of effort in college activities and learning gains of Chinese undergraduate students. First, male undergraduates interacted more with their teachers, engaged more in scientific activities, and gained more scientific experiences than their female counterparts. Previous research has shown the stereotype that pairs males with math-related disciplines and activities (Cvencek et al., 2014; Milesi et al., 2017), which further overestimates males’ performance and underestimates females’ performance (Reuben et al., 2014). For instance, regardless of gender and ethnicity, teachers ask male students more questions in the classroom, especially in math or science courses (Claire, 1995; Mizala et al., 2015). Male students received more attention from teachers not only verbally but also in nonverbal interactions, such as eye contact (Bennett and Lecompte, 1990). Therefore, male undergraduates, who built close relationships with teachers, persisted in science-and mathematics-based courses and majors (Davis and Young, 1982; Rimm-Kaufman et al., 2015). Additionally, this result may result from different learning styles between genders, which have been supported by Else-Quest et al. (2010), who found a dramatic gender difference in mathematics interests and attitudes: Males expressed a more positive attitude toward mathematics than females. Although numerous studies have found similar performance and abilities in mathematical and scientific fields between genders (Hyde et al., 2008; Lindberg et al., 2010; Snyder and Dillow, 2011), understanding female students’ attitudes, motivation, and learning styles in STEM-related activities are still needed lots of attention from faculty and scholars. These results inspire teachers to be aware of possible gender bias in teaching and learning, equally interact with undergraduates with different gender, encourage them to participate in scientific activities, and enhance their scientific experience. In addition, it also reveals the need to take into account the cognitive states of different students, to provide them with a more personalized learning experience, to stimulate their interest in learning, and create a student-centered learning environment based on the distinct pace of instruction that each student wants to receive, including through new approaches to digital education (Troussas et al., 2020).

Moreover, freshmen participated more in activities using campus facilities, while seniors spent more time on library activities, writing activities, and activities with faculty. Freshmen entered university with strong curiosity and enthusiasm for campus facilities and student organizations. While seniors were pressured to write theses, they had more opportunities to communicate with their teachers and search for literature in the library. In this light, faculties and undergraduates’ counsellors should facilitate freshmen to better adapt to college life, and encourage them to accumulate more writing experience and actively use the library. Lastly, undergraduates in provincial key high schools engaged more in diverse college activities. They gained more than those from district key high school or ordinary high schools, which verified Astin’s IEO (Input-Environment-Output) theory (Astin and Antonio, 2012) that students’ quality of effort in college activities was related to their previous educational background. Considering students with disadvantaged educational backgrounds, universities should provide various educational activities and support strategies from which disadvantaged students could benefit.

Meanwhile, gaps in learning experiences between Chinese undergraduates and American undergraduates were revealed. One important finding is that Chinese undergraduates communicated less with teachers and participated less in the course than their American counterparts. This result is in line with the work of den Brok et al. (2002) that Asian students rated higher on power distance or approach to authority in teacher’s interpersonal behaviors compared to white students, because respect for authority and centralized leadership is highlighted in Asian culture, and Asian students are more sensitive to this aspect. It may further affect their interaction with teachers. Apart from cultural differences, control of learning rather than supportive teaching remains the domain teaching mode in Chinese HEIs (Yin et al., 2016), making Chinese undergraduates have little communication with their teachers (Chen, 2009; Yang, 2015). This teaching model has, to some extent, led to a lack of initiative in the classroom among Chinese undergraduates, who usually participate less in class discussions and rarely try to apply what they have learned to other areas. Therefore, we suggest that Chinese teachers should conduct student-oriented teaching methods, increase classroom interaction, communicate with students, and encourage students to participate in classroom discussions. Furthermore, we found that Chinese undergraduates have lower learning gains in intellectual skills than their American counterparts. Specifically, Chinese undergraduates are effective writers and speakers but poor synthesizers of ideas. Based on this result, faculty should strengthen undergraduates writing training and encourage undergraduates to express their ideas in class actively. In other words, undergraduates should practice communicating their ideas through writing or speaking and improve their ability to integrate different ideas and information.

Compared to demographics, quality of effort in college activities contributed more to learning gains, with either strong or weak impact based on different activities engagement. This study found that student acquaintances were the strongest predictor of personal development, which supports that peer learning and collaborative learning were the key to success (Yeh, 2004) and affected undergraduates’ personal and social development (Kaufman and Creamer, 1991). Moreover, we found that experience with arts, music and theater was the best predictor of gains in general education, and science and quantitative activity was the best predictor of gains in science and technology. Pace (1984) has demonstrated these results: the extent to which students use cultural facilities (art, music, and drama) was related to their gains in general education; the quality of effort in science and technology activities was related to undergraduates’ understanding of scientific progress. Furthermore, this study showed that participation in activities with college facilities was the best predictor of vocational preparation and intellectual skill. One exploratory reason is that undergraduates could use college facilities to improve their knowledge and abilities of a profession and to gather relevant career information. Based on these results, it is recommended that universities and faculty provide more opportunities to promote communication and collaborative learning, offer more science and arts activities, and provide more robust campus facilities for undergraduates.

## Limitations and future research development

Although these findings based on large-scale data can be used as a reference for universities with similar characteristics (i.e., comprehensive and research-oriented), it may be difficult to generalize to other universities, such as teaching-oriented or private universities. It also should be noted that university B is characterized by liberal arts and sciences, especially in education. Future studies should investigate undergraduates’ experiences in different types of universities in China. In addition, this study mainly examines the relationship between undergraduates’ background, quality of effort in college activities, and learning gains. However, other factors that affect undergraduates’ learning gains, such as institutional environment and faculty teaching style, should be explored in the future. Meanwhile, adapted from CSEQ, this study mainly measures Chinese undergraduates’ quality in college activities by the frequency of participation in college activities. Future research could explore other aspects, such as undergraduates’ emotional or cognitive involvement. Finally, the measurements applied in this study are subjective scales. Although students’ self-reported data are widely used as indicators of learning experiences to improve the college environment and promote student development (Pike, 1995), future studies could use an objective scale for measuring those variables to provide a wider view.

## Conclusion

Based on a large-scale survey of a case Chinese university, this study explores how Chinese undergraduates experience college activities and what they gain, respectively. We found that Chinese undergraduates engaged more in college facilities and student acquaintances, and less in activities related to teachers, scientific and quantitative experience, and cultural activities. Significant gender, grade, and type of high school differences in Chinese undergraduates’ experiences were found. Compared with the U.S norms, Chinese undergraduates had less interaction with teachers, lower classroom engagement, and lower gains in general education, science and technology, and intellectual skills. The quality of effort in college activities significantly predicted learning gains. Different activities engagement affected learning gains strongly or weakly.

Adapting from CSEQ (Gonyea et al., 2003), the study validates the applicability of the CSEQ in the Chinese context, which offers the possibility of further use of the CSEQ in other similar contexts. Additionally, the study found that undergraduates’ quality of effort in college activities contributed more to learning gains than demographic variables, which responds to the controversy of existing studies about the influential mechanism and provides Chinese empirical evidence. Furthermore, the study innovatively conducts a comparative study to analyze the differences in learning experiences and gains between U.S. Norms and Chinese undergraduates, and explore underlying reasons for differences. These results cannot only help Chinese universities enhance undergraduates’ learning experiences and gains, promote educational and teaching reforms, improve university governance, and provide a reference for similar contexts to promote the overall development of students and improve the quality of higher education.

## Data availability statement

The original contributions presented in the study are included in the article, further inquiries can be directed to the corresponding author/s.

## Ethics statement

Ethical review and approval was not required for the study on human participants in accordance with the local legislation and institutional requirements. Written informed consent for participation was not required for this study in accordance with the national legislation and the institutional requirements.

## Author contributions

CS and JL contributed to the conception and design of the study. CS designed the study, analyzed the data, and drafted the manuscript. JL completed the introduction and literature review, guided data analysis and interpretation, and revised critically. RD organized data collection, and revised the manuscript. JM collected data collection and interpretation and reviewed the final version of the paper, which all authors approved. All authors contributed to the article and approved the submitted version.

## Funding

This work was supported by the National Office for Education Sciences Planning (Ministry of Education, the People’s Republic of China) under Grant (no. BIA190180).

## Conflict of interest

The authors declare that the research was conducted without any commercial or financial relationships that could be construed as a potential conflict of interest.

## Publisher’s note

All claims expressed in this article are solely those of the authors and do not necessarily represent those of their affiliated organizations, or those of the publisher, the editors and the reviewers. Any product that may be evaluated in this article, or claim that may be made by its manufacturer, is not guaranteed or endorsed by the publisher.
